# Short-Pulse Laser-Assisted Fabrication of a Si-SiO_2_ Microcooling Device

**DOI:** 10.3390/mi12091054

**Published:** 2021-08-30

**Authors:** Alexandros Mouskeftaras, Stephan Beurthey, Julien Cogan, Gregory Hallewell, Olivier Leroy, David Grojo, Mathieu Perrin-Terrin

**Affiliations:** 1 Aix Marseille University, CNRS, LP3, UMR7341, 13284 Marseille, France; david.grojo@univ-amu.fr; 2 Aix Marseille University, CNRS/IN2P3, CPPM, Marseille, France; beurthey@cppm.in2p3.fr (S.B.); cogan@cppm.in2p3.fr (J.C.); gregh@cppm.in2p3.fr (G.H.); oleroy@cppm.in2p3.fr (O.L.); mathieu.perrin-terrin@cppm.in2p3.fr (M.P.-T.)

**Keywords:** laser materials processing, microcooling, microfluidic device

## Abstract

Thermal management is one of the main challenges in the most demanding detector technologies and for the future of microelectronics. Microfluidic cooling has been proposed as a fully integrated solution to the heat dissipation problem in modern high-power microelectronics. Traditional manufacturing of silicon-based microfluidic devices involves advanced, mask-based lithography techniques for surface patterning. The limited availability of such facilities prevents widespread development and use. We demonstrate the relevance of maskless laser writing to advantageously replace lithographic steps and provide a more prototype-friendly process flow. We use a 20 W infrared laser with a pulse duration of 50 ps to engrave and drill a 525 μm-thick silicon wafer. Anodic bonding to a SiO_2_ wafer is used to encapsulate the patterned surface. Mechanically clamped inlet/outlet connectors complete the fully operational microcooling device. The functionality of the device has been validated by thermofluidic measurements. Our approach constitutes a modular microfabrication solution that should facilitate prototyping studies of new concepts for co-designed electronics and microfluidics.

## 1. Introduction

Microfluidic technology has enabled the fabrication of a vast number of miniaturized devices [1]. Major application domains include micro-chemistry [2], micro-analytical devices [3], biomedicine [4] and more recently the microelectronics industry [5]. Commonly used microfabrication techniques allow the production of submillimetre cross-sectional dimension channels buried within the bulk of a solid in which different types of fluids can be circulated. If the embedded channels are combined with an optical detection system, these microfluidic chips reach their full potential in terms of compactness, low reagent consumption and relative ease of fabrication. Transparent materials such as glass and polymers (polycarbonate, polystyrene, polydimethylsiloxane, etc.) are used in their fabrication. The above-mentioned features are major advantages for “lab-on-a-chip” style environments and can allow smaller miniaturization laboratories to perform prototyping operations similar to those in full-scale industrial counterparts.

In the important field of microelectronics, expanding interest comes with the continuously growing density of components in high-power computing chips, which causes challenges in heat extraction to maintain desired performance levels. While convective cooling may be sufficient for some applications, when highly integrated 3D electronic architectures are present fluidic cooling has the best performance [6]. Furthermore, the presence of microelectronics in highly demanding environments—such as detectors used in high-energy particle physics—adds extra constraints to the requirements for the cooling scheme. Such constraints include a low material budget and a minimal temperature difference between the heat source and the coolant [7]. An innovative, silicon-based microfluidic plate combining arrays of microchannels has already proved to be successful [8]. However, its fabrication process was based on lithography techniques, resulting in limited flexibility within the prototyping phase and the multiplication of fabrication steps. The tedious cycles inherent to the fabrication process also lead to an increased failure probability. These issues need to be addressed in order to allow widespread access to research and development, and democratize the use of microcooling in future consumer electronics.

Traditional silicon-based microfluidic device fabrication benefits from long-established techniques driven by the microelectronics industry. In a typical microfabrication process flow for a silicon-based microfluidic device the surface of silicon is first patterned, then bonded to another wafer to encapsulate the structures and finally the fluidic connections are added. Patterning of the silicon surface may be achieved by either dry (deep reactive-ion etching) or wet (KOH, etc.) etching. In dry etching, a plasma reacts with the surface of the material being etched leading to high aspect ratios and vertical walls. The etching rate lies within 2–30 μm/min [9]. In wet etching, either anisotropic or isotropic etching may be performed. In isotropic etching, generally rounded walls are created and the etching rate is in the range of 4–90 μm/min [9]. In anisotropic wet etching, one can create V-grooves with etching rates in the order of 1 μm/min [9]. In all cases, a masking layer is used that needs to be patterned according to the desired design. Furthermore, at this stage demanding environments are necessary, thus limiting the possibility for widespread usage. Once the silicon wafer is patterned, it can be either directly bonded to glass by anodic bonding or to silicon by silicon fusion bonding. Another possibility is to use adhesive bonding at the expense of poorer bonding strength. As a wafer can contain several microfluidic devices, it is mechanically diced to obtain the individual microfluidic chips. Finally, inlet and outlet fluid connectors can be fixed at the through holes either by mechanical clamping or by metallization and soldering.

Laser-processing can be used alternatively to pattern the surface [10] or the bulk [11] of silicon. The advantage of this technique is that a simple computer-aided design (CAD) is read by a laser scanning system and then transposed onto the sample surface. Thus, there is no need for mask fabrication specific for each operation (engraving, drilling, etc.) nor are any toxic chemicals involved—making it a relatively accessible fabrication method for small-to-medium scale technology providers. Over the years, several types of lasers have been used with a wavelength within the (linear) optical absorption spectrum of silicon, ranging from ultraviolet (excimer laser) to infrared (Nd:YAG, Yt:YAG lasers, etc.). Furthermore, the effect of pulse duration has been explored with shorter laser pulses (fs to ps) proving to provide better machining quality. While the choice of the appropriate laser is subject to many criteria (such as material removal rate, processing quality, investment, and operation costs, etc.), a good compromise between them can be found in using an infrared (1 μm) picosecond (10^−11^ s–10^−12^ s) laser. Indeed, picosecond pulse duration provides satisfactory processing quality in anticipation of the demanding surface quality requirement for anodic bonding, while keeping investment costs reasonable (compared to the choice of a femtosecond laser). The choice of the laser wavelength is based on our previous studies [12] showing maximum material removal rate at the wavelength of 1 μm for silicon.

The focus of this work is to explore laser-assisted surface patterning of silicon for rapid prototyping of a microcooling device based on a Si-SiO_2_ microfluidic plate. The usual steps of wet or dry etching are replaced by direct laser writing. While wet or dry etching techniques demand fabrication of complex and costly masks, direct maskless writing by laser gives the freedom of potentially writing any 2.5D processing pattern within the limits of aspect ratio and quality. We validate the different fabrication steps leading to the completion of a functional microcooling device prototype. Finally, we present thermo-hydraulic test results.

## 2. Materials and Methods

**Figure 1:**
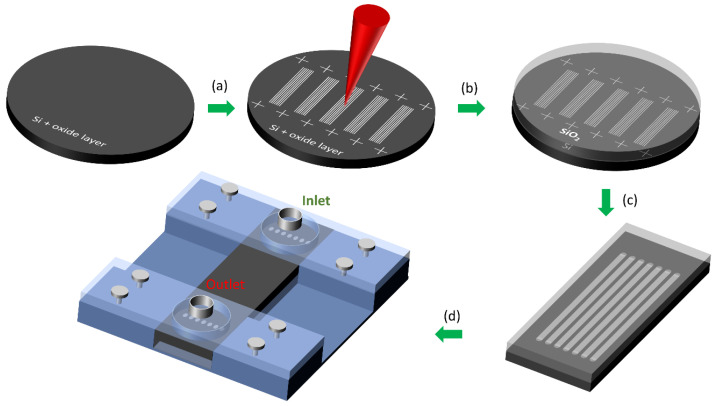
Fabrication process flow for producing Si-SiO_2_ microfluidic chips: (**a**) Surface patterning of Si by use of laser ablation, (**b**) Sample cleaning and anodic bonding to a borosilicate wafer for encapsulation, (**c**) Dicing of the individual chips and (**d**) Mechanical clamping of inlet/outlet connectors.

In this section, we present and detail the fabrication methods for producing Si-SiO_2_ microfluidic chips. The overall fabrication process flow is summarized in Figure 1. First, we use a silicon wafer with an oxide layer on the surface. The role of the oxide is to collect debris from processing and upon removal of this layer, leave a clean Si surface behind. Subsequent laser machining allows us to both engrave microchannels on the Si surface and drill through holes (Figure 1a). The same laser is also used to mark the surface of the sample with fiducial crosses that are used as position references for the rest of the process. Following the machining step we perform a chemical etch of the oxide layer to remove any laser ablation debris still present on the surface followed by a mechanical polishing step. We then perform anodic bonding between the machined Si wafer and a borosilicate glass wafer (Figure 1b). We use a diamond disc cutter to dice the microfluidic chips at the previously defined cross markings to obtain several chips per wafer (Figure 1c). Finally, the chip is positioned in a custom-made holder (Figure 1d) that allows inlet and outlet connectors to be firmly placed on top of the silicon surface over the laser-drilled holes.

We have used p-doped silicon test wafers with a <100> orientation and a resistivity within the 0.1–100 ohm.cm range. The wafers have been intentionally oxidized (to serve as sacrificial layer for debris removal) on their optically polished surface before the laser processing with an oxide layer thickness of 2 μm. The wafers had a diameter of 100 mm +/−0.5 mm and a thickness of 525 +/−25 μm.

**Figure 2:**
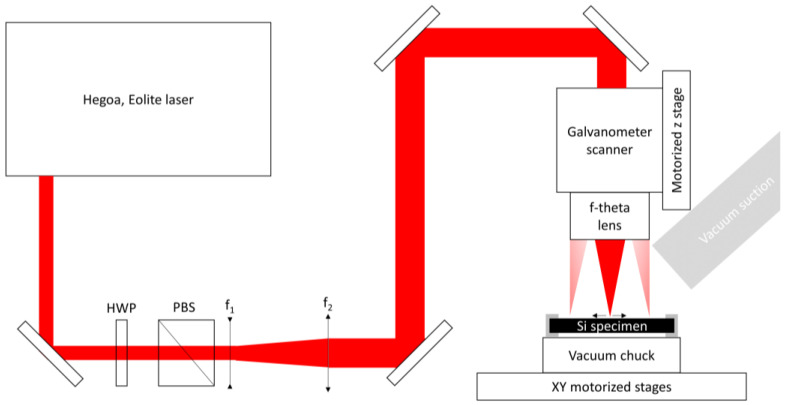
Laser processing experimental configuration for silicon engraving and drilling. The following acronyms are used, HWP: Half-wave plate, PBS: Polarizing beamsplitter cube.

To process our sample, we have used the home-built laser-machining platform illustrated in Figure 2. A picosecond fiber laser (Hegoa, Eolite, Pessac, France) was used, whose characteristics are summarized in Table 1. The laser beam power can be attenuated with the help of a half-wave plate and a polarizing beam-splitting cube. It is also possible to modify the pulse repetition rate by operating an internal acousto-optic modulator. The laser beam diameter is magnified by using a 2x beam expander composed of a diverging (*f*_1_ = −100 mm) and a converging (*f*_2_ = 200 mm) lens. The beam is then guided with dielectric mirrors to the entrance of a galvanometer scanner (Intelliscan 14, Scanlab, Puchheim, Germany) equipped with a 160 mm f-theta lens (Qioptic, Rhyl, United Kingdom). The laser beam at the focus has a diameter of ~35 μm measured by using the diameter-regression technique [13]. The laser focal plane is positioned with respect to the sample surface with the help of a Z-motorized linear translation stage (Newport, Irvine, CA, United States).

The sample is placed between annular metallic collars in what constitutes a home-made sample holder which allows easy manipulation of the wafer without handling it and provides an open-air space below the wafer rear surface. The latter has been shown to be an important advantage during the development of our process as it helps the evacuation of matter more easily during the laser drilling procedures. The sample holder is then fixed on an X-Y motorized linear translation stage (Newport, Irvine, CA, United States) to allow coarse positioning of the sample within the optical scanner field of view. A hose connected to the building’s air extraction system is placed close to the sample to aspirate and remove ablation debris.

All the above-mentioned equipment is controlled by a Labview program that allows coordination between X-Y-Z translation of the sample, operation of the laser’s optical shutter (acousto-optic modulator) and execution of the imported beam trajectory on the sample developed with the software provided by Scanlab (Puchheim, Germany).

Optical inspections and observation of the processing results were made using an optical microscope (Eclipse LV100ND, Nikon, Minato City, Japan) operated in both reflection and transmission mode. The engraved depth was measured using a confocal microscope (DCM 3D, LEICA, Weltzlar, Germany).

The microchannels are sealed by anodic bonding of a borosilicate wafer (Borofloat 33, Schott, Mainz, Germany) to the patterned silicon wafer. These operations are performed at the Centre for MicroNanoTechnology (CMi) at Ecole Polytechnique Fédérale de Lausanne (EPFL). After wafer cleaning and inspection, the thickness of the oxide layer was measured (Filmetrics, San Diego, CA, United States) and the layer removed. The wafer was then dipped for 18 min in buffered hydrofluoric acid (BHF: NH4F 40%, HF 50%, 7:1). Wafer inspection was performed after the oxide layer removal. A 60 s mechanical polishing step (E 460 CMP, Alpsitec, Saint-Martin-le-Vinoux, France)—using a steel pad without slurry—was then performed to further remove debris. The wafer was subsequently rinsed with deionized water and dried using a spinner. Following inspection, the silicon wafer was bonded (Süss SB6, Garching, Germany) to the borosilicate wafer. The bonding is performed at a temperature of 350 °C, at a vacuum pressure of 1000 mbar and with a pressure on the wafer stack of 150 kPA. A voltage of −1205 V is applied between the silicon and the borosilicate wafers and the current is monitored until it reaches 14% of the initial value. The wafer stack is finally diced using a circular saw (DAD-321, Disco, Tokyo, Japan) using the cross marks etched by laser and visible through the borosilicate wafer.

The wafer inspections and observations at CMi are performed with Bruker Dektak XT profiler (Billerica, MA, United States), Nikon LV150 microscope (Minato City, Japan), Nanospec AFT-6100 spectroscopic reflectometer (Nanometrics, Kanata, ON, Canada) and Zeiss Merlin scanning electron microscope (Jena, Germany).

**Figure 3:**
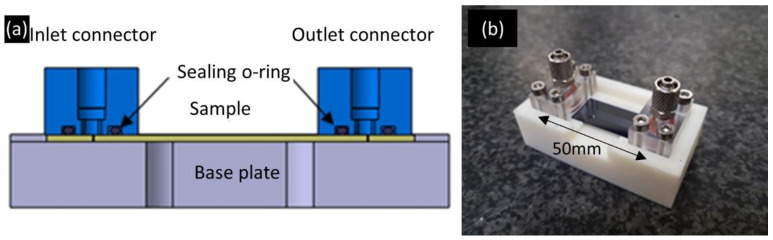
Schematic of the inlet and outlet connectors (**a**) and picture of the microcooling device assembled with the inlet and outlet system (**b**).

The functionality of the cooling device is tested by circulating water at a controlled temperature while heating the device with a kapton heater mimicking the power dissipated by a silicon-based charged particle detector. To circulate the cooling liquid inside the micro-cooling device, a system was manufactured in-house. This equipment is composed of a base plate in acrylonitrile butadiene styrene supporting the sample (Figure 3) onto which two connectors in clear polycarbonate are placed facing the 10 inlet/outlet holes drilled in the sample. These act as manifolds, covering all inlet and outlet holes and supplying each channel with a fluid at identical pressure. O-rings provide the sealing between the connectors and the sample. Each connector is tightened onto the base plate by 4 screws.

**Figure 4:**
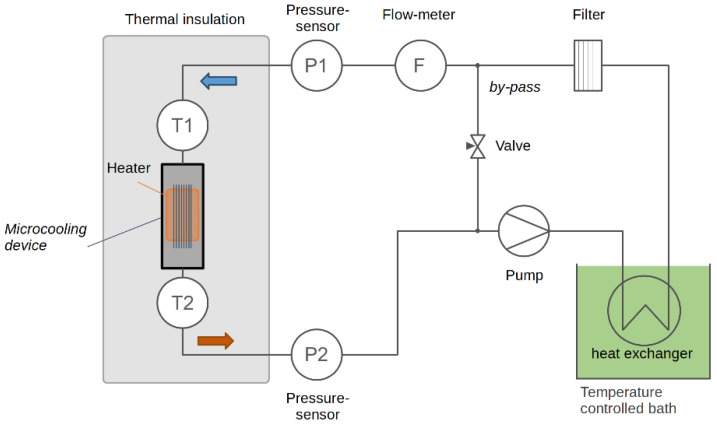
Schematic of the cooling circuit used to test the functionality of the microcooling device.

A schematic of the flow circuit is shown in Figure 4. A diaphragm pump (KNF Neuberger NF1.100 KTDCB, Balterswil, Switzerland) allowing a maximum flow of 1.2 L/min with a maximum pressure of 6 bar (g) circulates the water in a closed circuit of tubing with 4 mm inner diameter. In order to keep the temperature at the inlet of the microcooling device constant, a length of 50 cm of tubing is immersed in the bath of a temperature-controlled chiller. Special care is taken to prevent as far as possible the clogging of the micro-channel: deionized water is used and a filter (Classic Filter Co., Rochester, United Kingdom model 12.57.S1V fritted 316 stainless steel cartridge, 1 micron pore size: cartridge holder resistant to 7 bar (g)) to block possible sediments. To adapt the total flow to the pump operational range and vary the flow through the microcooling device, only a fraction—controlled by a by-pass valve—of the total flow passes through the microcooling device. The device is instrumented with PT100 temperature sensors and pressure sensors (RS-pro 7975046, Corby, United Kingdom) to measure the liquid pressure at its entrance and exit. A clamp-on ultrasonic flowmeter (Keyence FD-SX8 head with FD-XA1 controller, Osaka, Japan) measures the water flow rate.

**Figure 5:**
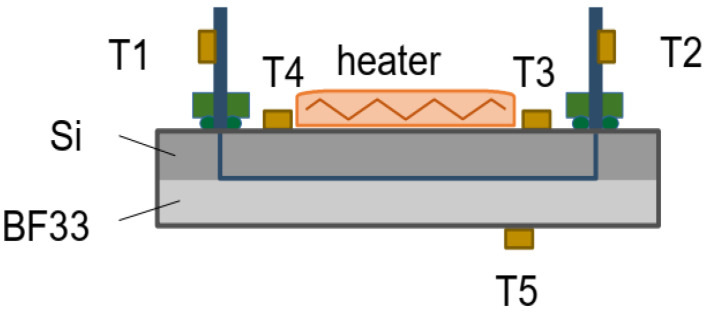
Schematic of showing the temperature sensors positioning on the cooling-device.

The position of the various temperature sensors are shown in Figure 5. Sensors T_1_ and T_2_ are placed on the inlet and outlet cooling pipes. T_3_ and T_4_ are glued on the surface of the silicon near the inlet and outlet of the device, respectively, while T_5_ is glued on the back glass cover. A kapton heater of (2 × 2) cm^2^, embedding a resistor of 18.8 Ω, is glued on the silicon side of the cooling device and can dissipate a power of up to 5.3 W at a maximum voltage of 10 V. The heater being larger than the device, the effective surface where the heat is dissipated in the silicon is only 2 cm^2^. The microcooling device as well as the portion of the circuit where the temperatures are measured are thermally insulated from the environment with low density foam. The ultrasonic flowmeter delivers a 4–20 mA analog signal proportional to flow rates up to 40 mL/min, which is converted to a voltage in the range 0–5 V through a resistor. The pressure sensors deliver analog signals between 0 and 5 V for pressures between 0 and 6 bar (g). These analog signals are digitized with a 12-bit ADC (Microchip MCP3208, Chandler, AZ, United States) and converted to 3.3 V levels using a 4-channel level converter (Adafruit BSS138, New York City, NY, United States). The resulting digital signals are read out on the SPI bus of a micro-computer with digital I/O capabilities (Raspberry Pi3 model B, Cambridge, United Kingdom). The PT100 temperature sensors are read-out using 4-wire connectivity into amplifiers equipped with an internal ADC (Adafruit MAX31865, New York City, NY, United States) on a second Raspberry SPI bus. The measurements are recorded using custom python-based data acquisition software.

## 3. Results

### 3.1. Laser Patterning

**Figure 6:**
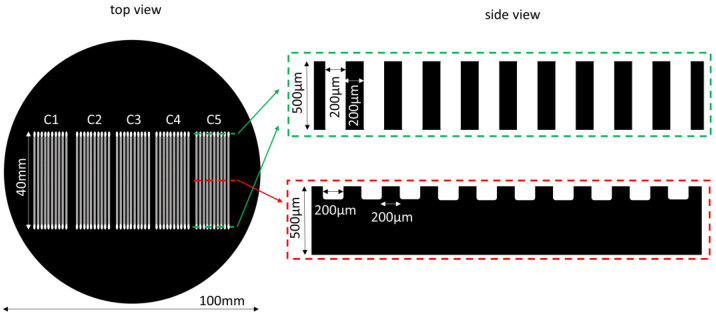
Design pattern for 5 microfluidic chips. Top view shows a panoramic view of the test pattern on the sample. Side view corresponds to transverse cuts at positions marked by dashed lines.

We have used our laser processing setup for subtractive manufacturing according to the design of Figure 6. The test pattern is composed of 5 stand-alone microfluidic chips (C1–C5), each composed of ten 70 μm deep microchannels of 40 mm length and 200 μm width. At their extremities lie through-holes of 200 μm diameter. The main focus in our application was to optimize the laser and scanning parameters in order to obtain satisfactory processing quality (minimization of recast layer on the edges) at high processing speed (in order to be competitive with other processing techniques).

**Figure 7:**
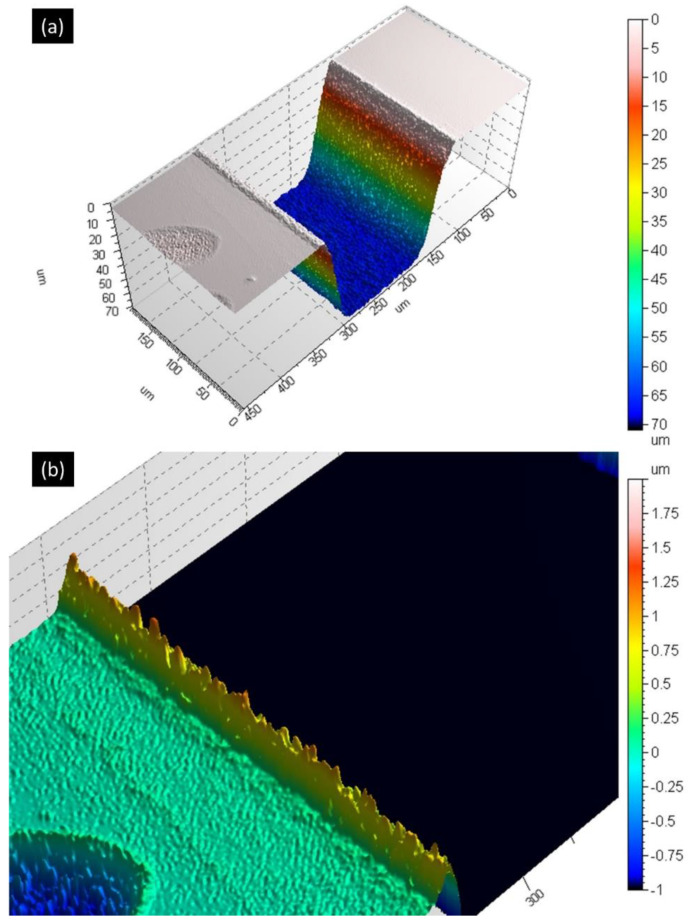
(**a**) Confocal microscope image of a portion of a laser-fabricated microchannel on the silicon surface. The width is ~200 μm and the maximum depth is ~70 μm, (**b**) Zoom-in on the edge of the laser-ablated channel.

Firstly, we searched for the optimal parameters for performing the laser engraving. Using a confocal microscope, we were able to evaluate the morphology of the laser-engraved structure. Figure 7a shows a portion of this laser-fabricated channel (32 mm long for this example) before oxide removal and mechanical polishing. The average roughness R_a_ was measured equal to ~530 nm and consequently has not been made the object of further optimization. Analysis of the surface at the edge of the microchannels (Figure 7b) showed presence of recast molten material over a maximum width of 10 μm with an average of 750 nm peak height. The ratio of the height of the recast molten material to ablated depth was of the order of ~1% owing to the use of a combination of low laser repetition rate and low pulse energy. This was among the main objectives as it is an important requirement to perform successful bonding afterwards.

For this example, we have used a previous version of our setup without the telescope of Figure 2 and thus with a beam diameter at the focus of 70 μm. We have used the following laser parameters: a pulse energy of 15 μJ (close to the maximum available energy) and a repetition rate of 200 kHz. The resulting laser peak fluency was 0.78 J/cm^2^, corresponding to just 1.1 times the threshold fluence for modification of the surface of silicon (F_th_ was found to be 0.7 J/cm^2^ using the Liu method [13]). This value is consistent with previous measurements [12]. The focus of the beam was carefully placed on the surface of the wafer by previously scanning the beam focus along the optical axis (z-scan). In all our experiments, in order to remove the material volume according to the design, we scan the beam along the given shapes (e.g., a circle for a hole, a rectangle for a trench). In the CAD file this is translated as hatching of the 2D shapes with line distance of 5 μm ensuring overlap between adjacent scanning lines. The pattern has to be repeated 350 times to reach the desired depth leading to a whole process lasting about 6 min to create a single channel as described in Figure 7. To improve the processing speed, we increased the applied laser fluence by reducing the spot size to 35 μm by use of the telescope of Figure 2. With a pulse energy of 20 µJ and an increase in the pulse repetition rate (500 kHz) we could reduce the number of repetitions of the laser scanning pattern necessary to reach the depth of 70 μm. With this new setting, only 63 repetitions were necessary resulting in the engraving of a 40-mm-long and 200-μm-wide channel in less than 3 min. These processing parameters have been retained for the fabrication of the prototype.

The next step in our experiment was to define the process for drilling 200 μm diameter through-holes in the 525 μm-thick silicon wafers. In our experiments, we noticed that—without the beam magnifier—attempts to laser-pierce the targets were futile due to insufficient laser fluence. Using the beam magnifier of Figure 2, we have extended the maximum available fluence to ~4 J/cm^2^. Furthermore, the beam focus was placed in the middle of the sample to allow maximum fluence distribution over the whole thickness. The pulse repetition rate was set at 255 kHz to limit any heat accumulation due to scanning over a small area for a long duration (due to the number of repetitions exceeding 1000).

**Figure 8:**
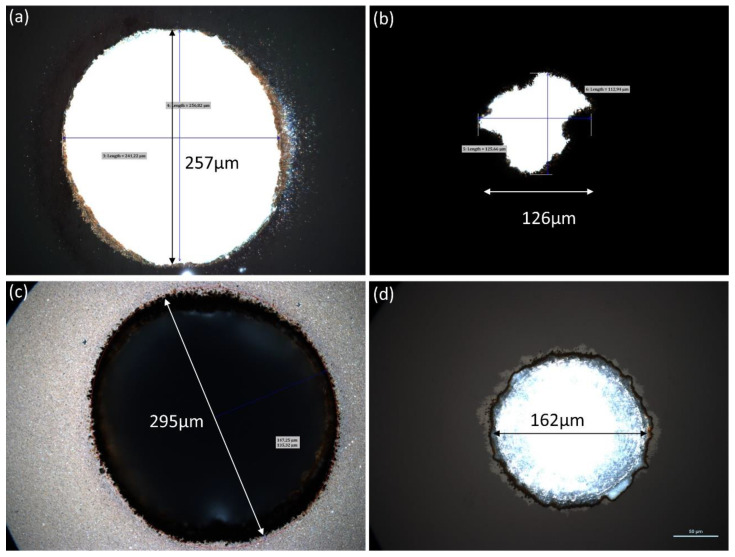
Microscope images of laser-drilled through holes. (**a**,**c**) correspond to the front surface and (**b**,**d**) to the rear surface. Different hole diameters are presented.

Given the fact that the targeted hole was wider than the size of the laser spot, scanning of the beam is needed to achieve the desired diameter. Several strategies were used including spiral scanning and linear hatching (with multiple orientations). The one that worked best was linear hatching of a >250 μm circle with an inter-hatchline distance of ~5 μm. To evaluate the traversing character of the holes we have used our microscope in transmission mode. Figure 8 illustrates microscope images of the front and rear faces of our sample following processing. The white color corresponds to light being efficiently transmitted through the hole. It is possible to pierce the wafer for hole diameters >250 μm. However, the hole diameter at the front surface plays a crucial role in the shape of the exit hole. To illustrate this, we show a hole with maximum input diameter of 257 μm in Figure 8a resulting in a 126 μm, poorly defined exit hole in Figure 8b. Increasing the diameter of the entry hole allowed us to obtain a well-defined circular hole at the exit. This is illustrated for a 295 μm-hole in Figure 8c resulting in a 162 μm exit hole in Figure 8d. These results show that in our experimental configuration a widening of the initially 200 μm-hole is necessary in order to have a reproducible and well defined through-hole. A ratio of 2.5:1 (depth to diameter) can be reliably reproduced.

An alternative to respect our initial design and to approach the target of a through-hole diameter of 200 μm was double-sided piercing. Firstly, we scanned the beam over a linear hatching pattern of a 160 μm-diameter circle. This laser scanning pattern was repeated 3000 times to evacuate enough volume to pierce through the sample. Next, the beam was scanned over 40 mm-long by 170 μm-wide hatched rectangle and repeated 63 times. Finally, we inverted the sample, and with precise positioning were able to scan the beam again over a linear hatching pattern of a 160 μm-diameter circle that overlaps with the holes created on the front surface. The main parameters for engraving and piercing are summarized in Table 2. The total processing time for a single microchannel with through holes was approximately 12.8 min.

**Figure 9:**
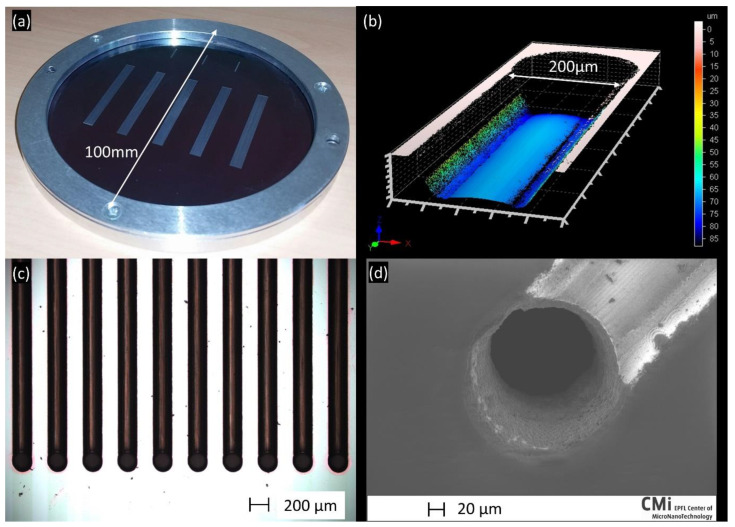
Results of laser-fabricated microchannels with through-holes at the extremities. (**a**) Photograph of the processed silicon wafer in its annular sample holder, (**b**) Confocal microscope image showing the morphology of a channel at its extremity, (**c**) Optical microscope image of the 10 microchannels with their through-holes at the bottom and (**d**) SEM picture of the hole and the channel.

In Figure 9a the silicon wafer is shown with five sets of microchannel arrays (ten microchannels per array) with through holes at the extremities. Figure 9b shows the 3D profile of a microchannel with a hole at the extremity. On average, the microchannels are 40 mm long and 205 ± 5 μm wide. Their depth over the whole length is 70 ± 4 μm. Tapering is observed as before (Figure 7) and the bottom is not flat but rather curved. There is a 10 μm difference in depth between the edges of the trench and the middle. The holes have an average diameter of 200 μm on the front surface and 230 μm at the rear surface. The holes have a bottleneck shape with the smallest diameter of 120 μm at the middle (along the Z-direction) of the sample. Figure 9c shows a microscope picture with an overview of ten microchannels with their corresponding holes at the extremity. Finally, Figure 9d shows a scanning electron microscope (SEM) picture of the hole and the microchannel. The time necessary to machine an array of ten microchannels such as those in Figure 9a is ~2 h.

### 3.2. Anodic Bonding of Si and SiO_2_ Wafers

**Figure 10:**
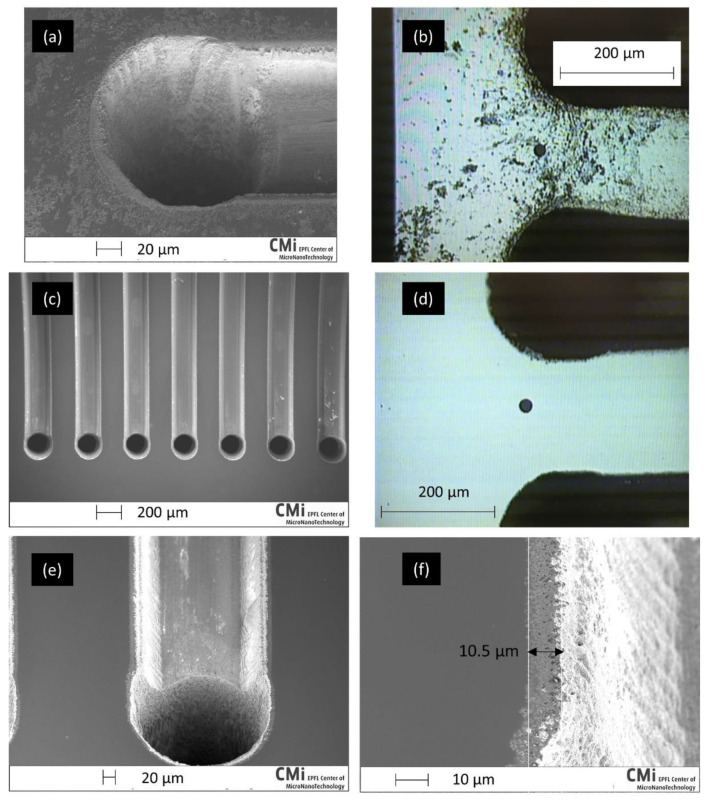
SEM and optical images of the wafer surface after oxide removal (**a**,**b**) and after the polishing (**c**–**f**). The back dot on (**b**,**d**) is an artefact from the observation device.

Inspection of the wafer surface at CMi revealed much debris around the etched area. The removal of the oxide layer reduced the amount of pollution but did not completely remove it, as shown in Figure 10a,b. As the quality of the anodic bonding strongly depends on the surface cleanliness, a mechanical polishing was further performed. This operation greatly improved the surface quality, as shown in Figure 10c–f. After polishing, surface defects remained only on a 10.5 μm wide area around the channel, as illustrated in Figure 10f. We attribute these defects to the combination a heat affected zone and the result of CMP performed on the recast molten material ridge present in Figure 7b.

**Figure 11:**
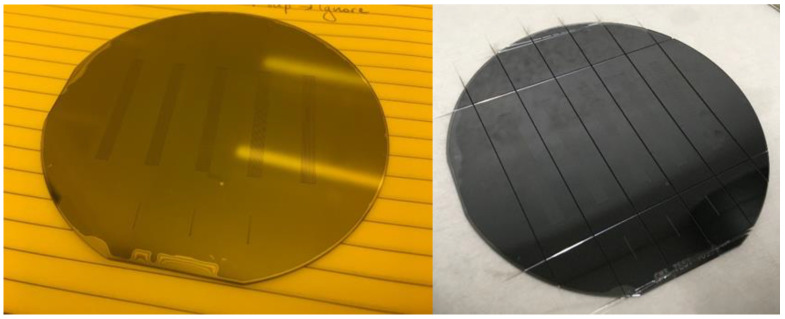
Wafer stack after bonding (**left**) and dicing (**right**). Some bonding defects are visible at the wafer edge and one in the inner region, all far from the channels.

The bonding of the wafer stack exhibited good quality with minor defects confined at the edges, with the exception of one stack where defects were also seen in a localized inner region of the wafer, fortunately far from any etched area, as shown in Figure 11 (left). The wafers were subsequently diced, as shown in Figure 11 (right).

### 3.3. Thermo-Hydraulic Measurements

**Figure 12:**
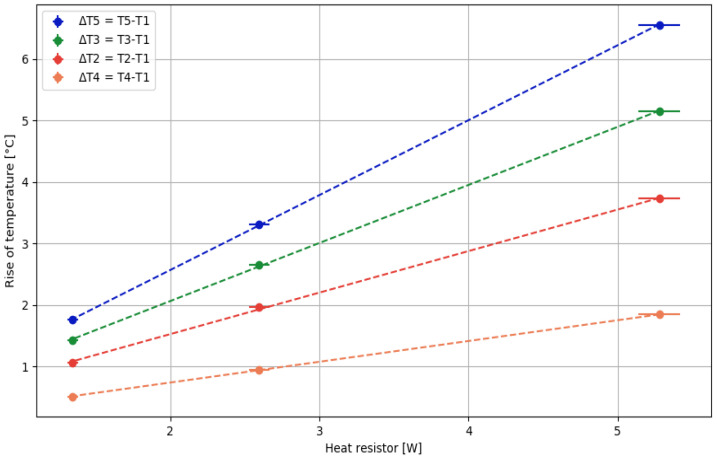
Measured temperature rises w.r.t. the input water temperature as a function of the power dissipated by the heater: ΔT2 (red) is the increase in the water temperature passing through the cooling device, ΔT5 (blue) is the difference between the temperature measured by the T5 sensor on the back of the device and the input water temperature. ΔT4 (orange) and ΔT3 (green) are the difference of temperature measured by the T4 and T3 sensors placed on the silicon side and the input water, respectively.

The functionality of the microcooling device was evaluated by circulating water at 20 °C, close to room temperature to minimize thermal losses between the setup and the environment. The flow rate was ~1.3 L/h, leading to a pressure drop inside the device—including the inlet and outlet manifolds—of around 5.5 bar. The temperatures of the water at the input and output of the device and those on its surface were recorded for different heater power dissipations. The observed temperature rises with respect to the temperature of the water at the inlet are shown as a function of the dissipated heat on Figure 12.

As expected, the rises are proportional to the dissipated heat and can be normalized to the dissipated power. The average rise of the water temperature inside the device (ΔT_2_ = ~0.7 °C/W) is compatible with the expectation given the water heat capacity (*C*_p_ = 4182 J·kg^−1^·K^−1^) and the flow rate. The highest temperature is observed on the back of the sensor (ΔT_5_) and corresponds to a rise of ~1.2 °C/W above the input water temperature. Lower rises are observed on the silicon side (ΔT_4_ = ~0.4 to ΔT_3_ = ~1 °C/W, depending on the sensor position). These values are in the ballpark of the typical temperature rises observed with this kind of device [8], showing that the microcooling device is functional.

## 4. Discussion

In Section 3.1, we show that it is possible to engrave silicon from the surface and to create microfluidic channels. While it is possible to obtain satisfactory processing quality (flat profile and low roughness as seen in Figure 7) with fluences of the order of F_th_, raising the fluence to 4 × F_th_ results in a concave curved bottom (Figure 9b) of these channels. It is a general trend to sacrifice processing quality with increasing laser fluences [14] but in this particular case, the over-ablated edges may be due to the reflection of the scanning laser beam on the tapered sidewalls and redirection towards the edges. In our case the morphology of the ablated structures at the bottom was not of primary importance. However, this problem can be most probably solved by adding a “gentle” (close to F_th_) ablation step at the end of the process to flatten the central part and even the profile of the trench. Furthermore, the channel of Figure 7 has a trapezoid shape with a width of ~200 μm at the surface and ~108 μm at the achieved depth of ~69 μm. This type of tapering is common in laser processing and arises mainly from the use of gaussian beams. An alternative is to use “top hat” profile laser beams or “trepan” scanning schemes.

The tapering effect becomes problematic when trying to drill holes with a high depth-to-diameter ratio, as in those of our design in Figure 6. Indeed, as previously mentioned, laser processing produces tapered structures which at a certain point become conical. This has as a result an increase in the apparent surface on which the laser spot is incident and a resulting lowered fluence, below that necessary for ablating the target. Thus, for a given available laser fluence there is a limited accessible aspect ratio. Finally, the entry holes in Figure 8a,b have a slightly elliptical shape which could be due to the scanning strategy (vertical hatchlines). Crossing the hatchlines should result in a less elliptical shape. In this article, we focus on the feasibility of the process flow and consider most of these aspects to presently be of minor importance.

One of the most important features in the fabrication of a microfluidic device is the processing speed. From our results we can deduce the average material removal rate defined as the ratio of the total machined volume to the time consumed. In the case of engraving of a 40-mm-long, 200-μm wide and 70-μm deep microchannel we have measured a rate of 0.2 mm^3^/min. An equivalent laser etch rate would be in that case equal to 24 μm/min. This value places the laser processing method within the highest values for dry etching and within the average of most wet etching techniques (see Section 1). It is evident that when more than one microchannel needs to be patterned then the laser etch rate quickly drops and it is inversely proportional to the number of the microchannels. The average material removal rate in the case of drilling a hole of 200 μm-diameter in 0.525 mm thick quickly drops within one order of magnitude compared to the engraving application, making the laser processing overal less competitive than dry or wet etching techniques for large production. However, at the prototyping phase where small numbers of devices need to be produced and tested, maskless laser writing can certainly be beneficial. Optimization of the processing speed can be easily achieved by either of the following pathways:

▪ Increase the pulse fluence [12];
▪ Use water assisted laser ablation [15] for more efficient drilling (a two-fold processing speed increase can be achieved if the need for double-sided ablation is removed);
▪ Use of bursts of laser pulses [16];
▪ Work with shorter pulses [17]. We may expect for instance a seven-fold increase in the processing speed by using a 400 fs pulse duration [18].

With the advent of parallel laser processing (through the use of spatial light modulators or multiple laser scanning heads) direct laser writing could soon make its entry into midscale production.

More important breakthroughs can be imagined if the anodic bonding step is suppressed. It has been shown that it is possible to use the laser to weld a patterned glass plate to an encapsulating glass plate, resulting in a functional microfluidic device [19]. With the more recent possibilities for silicon 3D writing [20,21], direct monolithic integration of microfluidic channels can be imagined. Since such techniques are already applicable in the case of transparent microfluidics [22] one can foresee fully integrated microcooling chips in the future with very modest sample preparation and no need for clean room facilities, thus considerably reducing the fabrication costs.

## 5. Conclusions

We report on an alternative microfabrication process flow aiming at fast prototyping for producing Si-SiO_2_ microcooling devices. We replace traditional lithography-based techniques combined with wet and/or dry etching with laser ablative material processing. A 20 W, 50-ps laser emitting at 1.05 μm is used for the demonstration of through-silicon via drilling and surface engraving in 0.525 mm-thick silicon. The compatibility of this technique with anodic bonding is demonstrated by sealing the patterned silicon surface with a glass wafer. As a final step, the functionality of the cooling device has been validated using a thermal setup reproducing typical operational conditions of a micro-chip dissipating a few W/cm^2^. Promising thermal performance (increase in temperature of a few °C/W/cm^2^) has been observed, directly supporting the potential of microcooling device technologies.

## Figures and Tables

**Table 1:** Laser characteristics.

Wavelength	Pulse Duration	Repetition Rate	Max Average Power	Max Energy Per Pulse
1030 nm	50 ps	1 MHz	20 W	20 μJ

**Table 2:** Laser and scanning parameters for silicon patterning.

Application	Pulse Duration	Focus Position	Repetition Rate	Laser Fluence	Scanning Speed	Repetitions
Engraving	50 ps	Surface	500 kHz	4 J/cm^2^	1 m/s	63
Drilling		Middle	250 kHz			3000

## Data Availability

The data presented in this study are available on request from the corresponding author.

## References

[1] Whitesides G.M. (2006). The origins and the future of microfluidics. Nature.

[2] Elvira K.S., Solvas X.C.I., Wootton R.C.R., Demello A.J. (2013). The past, present and potential for microfluidic reactor technology in chemical synthesis. Nat. Chem..

[3] Crowley T.A., Pizziconi V. (2005). Isolation of plasma from whole blood using planar microfilters for lab-on-a-chip applications. Lab. Chip.

[4] Niculescu A., Chircov C., Alexandra C., Grumezescu A.M. (2011). Fabrication and Applications of Microfluidic Devices: A Review. Int. J. Mol. Sci..

[5] Van Erp R., Soleimanzadeh R., Nela L., Kampitsis G., Matioli E. (2020). Co-designing electronics with microfluidics for more sustainable cooling. Nature.

[6] Wang S., Yin Y., Hu C., Rezai P. (2018). 3D integrated circuit cooling with microfluidics. Micromachines.

[7] Mapelli A., Petagna P., Howell K., Nuessle G., Renaud P. (2012). Microfluidic cooling for detectors and electronics. J. Instrum..

[8] Romagnoli G., Feito D.A., Brunel B., Catinaccio A., Degrange J., Mapelli A., Morel M., Noel J., Petagna P. (2015). Silicon micro-fluidic cooling for NA62 GTK pixel detectors. Microelectron. Eng..

[9] Iliescu C., Taylor H., Avram M., Miao J., Franssila S. (2012). A practical guide for the fabrication of microfluidic devices using glass and silicon. Biomicrofluidics.

[10] Phillips K.C., Gandhi H.H., Mazur E., Sundaram S.K. (2015). Ultrafast laser processing of materials: A review. Adv. Opt. Photonics.

[11] Verburg P.C., Smillie L.A., Römer G.R.B.E., Haberl B., Bradby J.E., Williams J.S., Huis in ’t Veld A.J. (2015). Crystal structure of laser-induced subsurface modifications in Si. Appl. Phys. A.

[12] Sikora A., Grojo D., Sentis M. (2017). Wavelength scaling of silicon laser ablation in picosecond regime. J. Appl. Phys..

[13] Liu J.M. (1982). Simple technique for measurements of pulsed Gaussian-beam spot sizes. Opt. Lett..

[14] Momma C., Chichkov B.N., Nolte S., Von Alvensleben F., Tünnermann A., Welling H., Wellegehausen B. (1996). Short-pulse laser ablation of solid targets. Opt. Commun..

[15] Butkus S., Alesenkov A., Paipulas D., Gaižauskas E., Melninkaitis A., Kaškelyte D., Barkauskas M., Sirutkaitis V. (2015). Analysis of the micromachining process of dielectric and metallic substrates immersed in water with femtosecond pulses. Micromachines.

[16] Kerse C., Kalaycloĝ Lu H., Elahi P., Çetin B., Kesim D.K., Akçaalan Ö., Yavaş S., Aşlk M.D., Öktem B., Hoogland H. (2016). Ablation-cooled material removal with ultrafast bursts of pulses. Nature.

[17] Ostendorf A., Kamlage G., Klug U., Korte F., Chichkov B.N. (2005). Femtosecond versus picosecond laser ablation. Proc. Photon Process. Microelectron. Photonics IV.

[18] Hodgson N., Heming S., Steinkopff A., Haloui H., Lee T.S. Ultrafast Laser Ablation at 1035 nm, 517 nm and 345 nm as a Function of Pulse Duration and Fluence. Proceedings of the Lasers in Manufacturing Conference.

[19] Wlodarczyk K.L., Carter R.M., Jahanbakhsh A., Lopes A.A., Mackenzie M.D., Maier R.R.J., Hand D.P., Maroto-Valer M.M. (2018). Rapid laser manufacturing of microfluidic devices from glass substrates. Micromachines.

[20] Chanal M., Fedorov V.Y., Chambonneau M., Clady R., Tzortzakis S., Grojo D. (2017). Crossing the threshold of ultrafast laser writing in bulk silicon. Nat. Commun..

[21] Chambonneau M., Wang X., Yu X., Li Q., Chaudanson D., Lei S., Grojo D. (2019). Positive- and negative-tone structuring of crystalline silicon by laser-assisted chemical etching. Opt. Lett..

[22] Sugioka K., Cheng Y. (2012). Femtosecond laser processing for optofluidic fabrication. Lab. Chip.

